# Primary cilia: a novel research approach to overcome anticancer drug resistance

**DOI:** 10.3389/fmolb.2023.1270639

**Published:** 2023-10-02

**Authors:** Kyung Ho Lee

**Affiliations:** ^1^ Chemical Biology Research Center, Korea Research Institute of Bioscience and Biotechnology (KRIBB), Ochang-eup, Republic of Korea; ^2^ Department of Bio-Molecular Science, KRIBB School of Bioscience, University of Science and Technology (UST), Daejeon, Republic of Korea

**Keywords:** primary cilia, ciliogenesis dynamics, anticancer drug, resistance, chemotherapy, cancer

## Abstract

Primary cilia are cellular organelles that consist of a microtubule skeleton surrounded by a membrane filled with cell signaling receptors. Many studies have shown that primary cilia are cellular antennas, which serve as signaling hubs and their assembly and disassembly are dynamically regulated throughout the cell cycle, playing an important role in regulating cellular homeostasis. Aberrant control of primary cilia dynamics causes a number of genetic disorders known as ciliopathies and is closely associated with tumorigenesis. Anticancer drug resistance is a primary cause of chemotherapy failure, although there is no apparent remedy. The recent identification of a relationship between anticancer drug resistance and primary ciliary dynamics has made primary cilia an important target subcellular organelle for overcoming anticancer drug resistance. Therefore, the research on primary ciliary dynamics may provide new strategies to overcome anticancer drug resistance, which is urgently needed. This review aims to summarize research on the relevance of primary cilia and anticancer drug resistance, as well as future possibilities for research on overcoming anticancer drug resistance utilizing primary cilia dynamics.

## 1 Introduction

Cancer is the fifth leading cause of death worldwide, but unfortunately, treatment success rates have not improved significantly over the past decades due to various limitations (Holohan et al., 2013; Alfarouk et al., 2015). These constraints include cancer cells developing drug resistance, drug toxicity, and cancer cell heterogeneity (Gottesman, 2002; Gottesman et al., 2002; Housman et al., 2014; Wang et al., 2019). Among these, the acquisition of drug resistance by cancer cells is one of the most fundamental factors leading to chemotherapy failure. Despite dedicated efforts by researchers, finding a way to overcome drug resistance remains elusive.

Chemical anticancer drugs, which have been used since the 1940s, have demonstrated some effectiveness against cancer, but their impact is limited by their tendency to harm normal cells and the development of resistance in cancer cells (Barinaga, 1997; Druker et al., 2001; Gottesman, 2002). The recent development of targeted anticancer drugs has significantly reduced the risk of harming normal cells, yet a definitive solution to cancer drug resistance remains elusive. Additionally, cancer immunotherapy, a promising next-generation treatment that harnesses the patient’s immune system, still faces limitations in inducing resistance and does not directly address cancer drug resistance (Said and Ibrahim, 2023). Therefore, understanding the fundamental mechanisms behind drug resistance and developing direct strategies to overcome it is necessary. By unraveling these underlying mechanisms, we can pave the way for more effective approaches to combat drug resistance and improve cancer treatment outcomes.

To date, research on anticancer drug resistance has focused on the mechanisms of gene expression regulation and gene mutation, with no focus on the identification of key cellular organelles that regulate anticancer drug resistance or the modulation of these organelles to overcome anticancer drug resistance. For cell signaling, primary cilia are known as cellular antennas/transmitters (Goetz and Anderson, 2010; Seeley and Nachury, 2010). Previously, the function of primary cilia has not received much attention; nevertheless, the role of primary cilia as a hub for cell signaling has been recognized (Davenport and Yoder, 2005; Seeley and Nachury, 2010). Primary cilia are receiving interest as a cellular organelle that might transcend the constraints of present illness therapies since they play a vital role in controlling cell homeostasis and have been connected to several human diseases (ciliopathy, cancer, etc.) (Badano et al., 2006; Han et al., 2009; Seeley et al., 2009; Wong et al., 2009; Davis and Katsanis, 2012; Snedecor et al., 2015). Primary cilia have been widely researched in the development of cancer, and the relevance of primary cilia dynamics to the development of anticancer drug resistance has recently been reported (Jenks et al., 2018; Kyun et al., 2020; Kim et al., 2022). Therefore, we will summarize the primary cilia studies related to anticancer drug resistance in this review and propose a new approach to overcome anticancer drug resistance utilizing primary cilia dynamics.

## 2 Primary cilia

**FIGURE 1:**
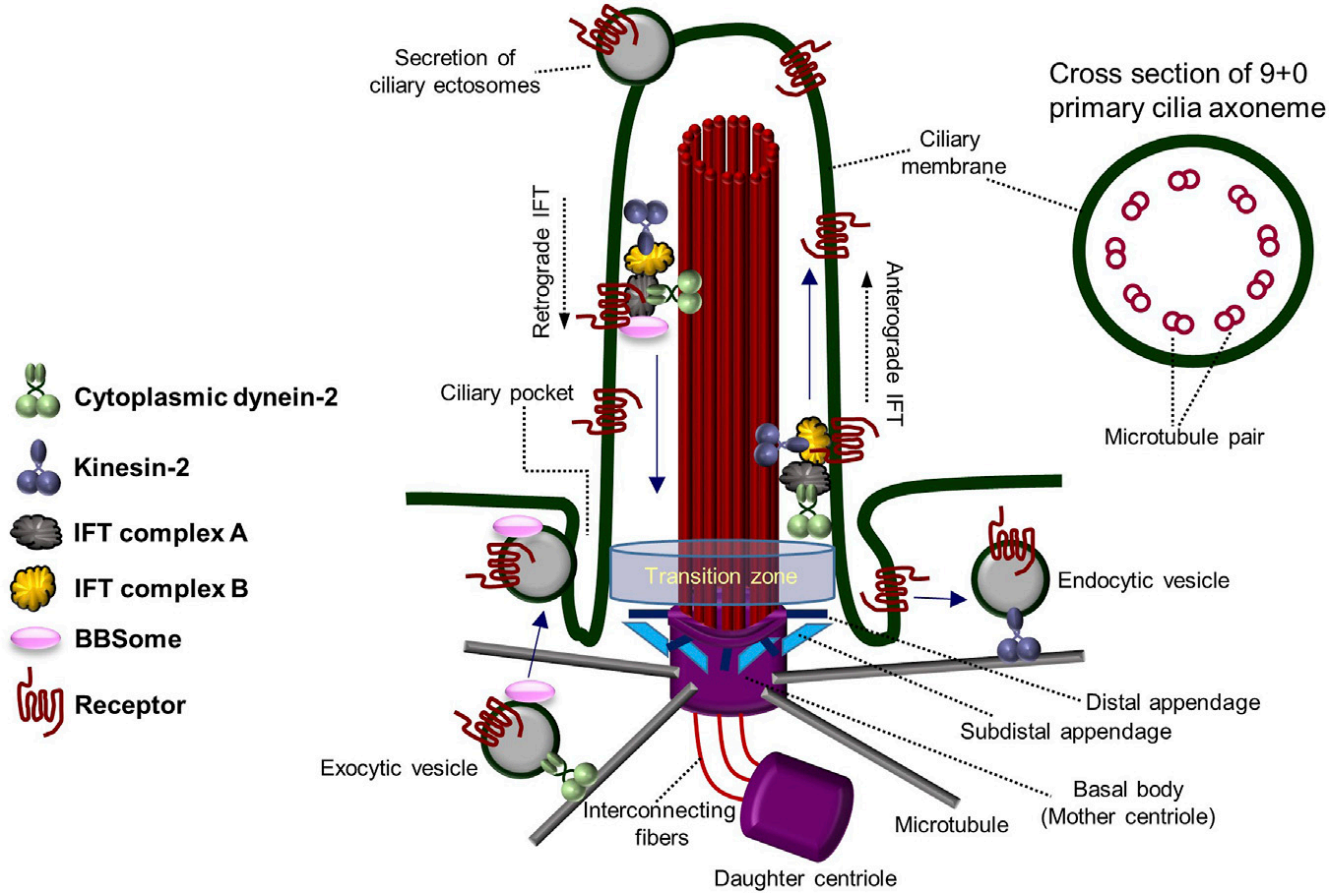
Architecture and components of the primary cilium. The overall structure and components of the primary cilium and the transport system within the primary cilium. Primary cilia are formed from the basal body (mother centriole) and have an axoneme consisting of “9 + 0” microtubule pairs. Figure reproduced from Ref. (Perkins et al., 1986; Afzelius, 2004; Davenport and Yoder, 2005; Fisch and Dupuis-Williams, 2011; Ke and Yang, 2014; Pedersen et al., 2016).

The primary cilium functions as a hub of cell signaling, comprising a microtubule skeleton and a membrane enriched with cell signaling receptors (Goetz and Anderson, 2010; Seeley and Nachury, 2010). Unlike the more widely known motile cilia, primary cilia are not self-motile; only one primary cilium is formed in a cell, and they have a dynamic assembly/disassembly cycle (primary cilia dynamics) where they are formed in the mother centriole during the G_0_/G_1_ cell cycle stage and then reabsorbed as they enter mitosis (Dawe et al., 2007; Pearson et al., 2007; Goetz and Anderson, 2010; Seeley and Nachury, 2010; Lee, 2020). The non-motility of primary cilia is caused by the structure of the axoneme, which has 9 + 0 morphology with nine microtubule doublets on the outside and no pair of microtubule singlets in the center, as well as the absence of structures that provide motility, such as dynein arms (Figure 1) (Perkins et al., 1986; Afzelius, 2004; Davenport and Yoder, 2005; Fisch and Dupuis-Williams, 2011; Ke and Yang, 2014; Pedersen et al., 2016). Motile cilia, on the contrary, possess 9 + 2 axonemal microtubule structure with a motile dynein arm. Due to their lack of motility and the fact that they are not permanent structures within the cell (Porter and Sale, 2000; Davenport and Yoder, 2005; Ke and Yang, 2014; Pedersen et al., 2016), primary cilia have been regarded as evolutionary vestige organs until recently.

In the 2000s, polycystin-1 and polycystin-2 proteins (mechanosensory complex), encoded by the causative genes PKD1 and PKD2 of polycystic kidney disease (PKD) (Pazour et al., 2002; Yoder et al., 2002; Masyuk et al., 2014), a genetic disorder, gained attention due to their specific presence in the primary cilia of renal tubular epithelial cells (Guay-Woodford, 2003; Davenport and Yoder, 2005). It has been reported that Tg737 mutant mice, the mouse/human homolog of Intraflagellar Transport 88 (IFT88) in *Chlamydomonas*, form shorter primary cilia than normal mice and cause lethality in PKD mice (Pazour et al., 2000; Haycraft et al., 2001; Taulman et al., 2001; Davenport and Yoder, 2005). Additionally, the suggestion that Bardet–Biedl Syndrome (BBS) may result from primary ciliary abnormalities also highlights the importance of primary cilia (Davenport and Yoder, 2005; Badano et al., 2006; Nachury et al., 2007). In addition to the characteristics distinguishing primary cilia from motile cilia, motile cilia are present in cells specialized for a particular function, whereas primary cilia are present in almost all animal epithelial cells (Perkins et al., 1986; Porter and Sale, 2000; Afzelius, 2004; Davenport and Yoder, 2005; Fisch and Dupuis-Williams, 2011; Ke and Yang, 2014; Pedersen et al., 2016). Since it disassembles as the cell cycle progresses, primary cilia have received less attention in the past. However, recent research suggests that primary ciliary dynamics may play an important role in fine-tuning cellular homeostasis, leading to the exploration of the delicate regulatory mechanisms associated with them. The assembly and disassembly of primary cilia are regulated by various cell signaling-related factors, but much remains to be understood (Lee, 2020). Primary cilia formation and degradation are influenced by intracellular signaling molecules and external factors. Hedgehog (Hh) signaling and canonical Wnt signaling have been reported to be involved in primary cilia assembly (Huangfu et al., 2003; Goetz and Anderson, 2010; Dafinger et al., 2011; Qin et al., 2011; Rix et al., 2011; Kyun et al., 2020; Lee, 2020), while platelet-derived growth factor (PDGF) signaling and noncanonical Wnt signaling are associated with primary cilia disassembly (Schneider et al., 2005; Pugacheva et al., 2007; Lee et al., 2012; Kim et al., 2021). Primary cilia are known to possess multiple cell signaling receptors on their surface, transmitting various cell signals into the cell. Additionally, dynamic vesicle-like secretion by mechanical fluid-shear stress occurs at the tip of the primary cilium, and secretion and absorption of vesicles occurs in the ciliary pocket near the lower basal body (Hogan et al., 2009; Chavez et al., 2015; Garcia-Gonzalo et al., 2015; Jensen et al., 2015; Pedersen et al., 2016; Nager et al., 2017; Phua et al., 2017; Mohieldin et al., 2020). These features allow primary cilia to precisely regulate cell signaling and abnormalities in these functions are suggested to contribute to many human diseases.

Genetic diseases caused by abnormalities in the regulation of cilia formation are collectively referred to as ciliopathies. These conditions include Bardet–Biedl syndrome (Davenport and Yoder, 2005; Badano et al., 2006; Nachury et al., 2007), Joubert syndrome (Badano et al., 2006; Davis and Katsanis, 2012), Meckel-Gruber syndrome (Badano et al., 2006; Sang et al., 2011), PKDs (Nonaka et al., 1998; Davenport and Yoder, 2005; Badano et al., 2006), and nephronophthisis (NPHP) (Davenport and Yoder, 2005; Badano et al., 2006; Sang et al., 2011), which share some symptomatic similarities. Common features observed in ciliopathies are developmental anomalies of the cerebellum and brain stem, kidney disease, retinal degeneration, loss of smell, polydactyly, obesity, and intellectual disability. More than 50 ciliopathy-related genes have been identified to date, almost all of which are located in the primary ciliary basal body (Reiter and Leroux, 2017). While the study of primary cilia initially gained attention in the context of rare genetic diseases like ciliopathy, it has recently received increasing attention due to its association with the development of cancer.

## 3 Primary cilia and cancer

Recently, researchers have recognized the critical role of primary cilia in tumorigenesis. However, it has been reported that the function of primary cilia in tumorigenesis and cancer metastasis can vary depending on the specific type of cancer and the cell type present in each organ. Therefore, there are conflicting reports on the association between cancer and primary cilia, and dual functions of primary cilia in cancer formation (mediating or suppressing tumorigenesis) have been reported (Eguether and Hahne, 2018; Liu et al., 2018; Wang et al., 2021). This variability makes primary cilia a potential biomarker for tumorigenesis, but their role must be carefully considered as either promoting or inhibiting tumorigenesis. Therefore, the precise role of primary cilia in tumorigenesis for each cancer type remains to be determined by further studies. However, based on the existing research, primary cilia deficiency has been observed in the majority of cancers, with a few exceptions (Higgins et al., 2019). Fewer primary cilia were observed in various carcinomas, such as glioblastoma (Yang et al., 2013; Moser et al., 2014; Sarkisian et al., 2014; Loskutov et al., 2018), melanoma (Snedecor et al., 2015), pancreatic tumors (Bailey et al., 2009; Seeley et al., 2009; Deng et al., 2017; Kobayashi et al., 2017), prostate cancer (Hassounah et al., 2013), ovarian cancer (Egeberg et al., 2012), colon cancer (Medema and Vermeulen, 2011; Rocha et al., 2014), breast cancer (Yuan et al., 2010; Menzl et al., 2014; Nobutani et al., 2014), medulloblastoma (Wechsler-Reya and Scott, 1999; Spassky et al., 2008; Han et al., 2009; Barakat et al., 2013), and renal cancer (Basten et al., 2013; Dere et al., 2015; Harlander et al., 2017; Kobayashi et al., 2017), compared to normal tissue.

Various dysregulations of primary cilia-related genes have been found in primary cilia-deficient carcinomas. In cholangiocarcinoma cases lacking primary cilia, inhibition of HDAC6, a primary cilia disassembly factor, restored primary cilia formation and inhibited cholangiocarcinoma growth (Peixoto et al., 2020). The *VHL* gene has been reported to be closely related to primary ciliogenesis and associated with the development of clear cell renal cell carcinoma (ccRCC) (Esteban et al., 2006; Arjumand and Sultana, 2012). Loss of primary cilia has been observed in ccRCC patients, and the re-expression of VHL protein in ccRCC carcinomas restores primary ciliogenesis (Esteban et al., 2006; Arjumand and Sultana, 2012; Basten et al., 2013). Furthermore, primary cilia-deficient renal and pancreatic cell carcinomas were not linked to the induction of primary cilia deficiency through increased cell proliferation, suggesting a direct connection between primary cilia deficiency and carcinogenesis (Seeley et al., 2009; Yuan et al., 2010). However, there are also studies showing that primary cilia regulate cell cycle progression or cell proliferation (Plotnikova et al., 2008; Goto et al., 2013; Khan et al., 2016; Jamal et al., 2020). In ovarian cancer cells, inhibition of primary ciliogenesis by perturbation of hedgehog signaling has been reported (Egeberg et al., 2012). Overexpression of Aurora A kinase (AurA), a primary ciliary disassembly factor (Pugacheva et al., 2007), in the ovarian surface epithelium leads to increased centrosomal AurA and disrupted hedgehog signaling, causing dysregulation of ovarian cell function and inducing tumorigenesis (Egeberg et al., 2012). This indicates that AurA overexpression inhibits Hh signaling, which in turn promotes primary ciliary disassembly and eventually induces ovarian cancer. In pancreatic cancer cells, and pancreatic intraepithelial neoplasia (PanIN) lesions from pancreatic ductal adenocarcinoma (PDAC), strong inhibition of primary cilia formation has been observed (Seeley et al., 2009). However, contrary to these findings, primary cilia were observed in approximately 25% of PDAC patients’ cancer cells, and patients with primary cilia showed higher rates of lymph node metastases (Emoto et al., 2013). These observations indicate a connection between primary cilia and tumorigenesis, but the impact of primary cilia on cancer may vary depending on the type of cancer and the tumor stage. Thus, while the importance of primary cilia in cancer research is evident, further work is needed to explore primary cilia-related signaling and its relevance to different types of cancer.

## 4 Anticancer drug resistance

**FIGURE 2:**
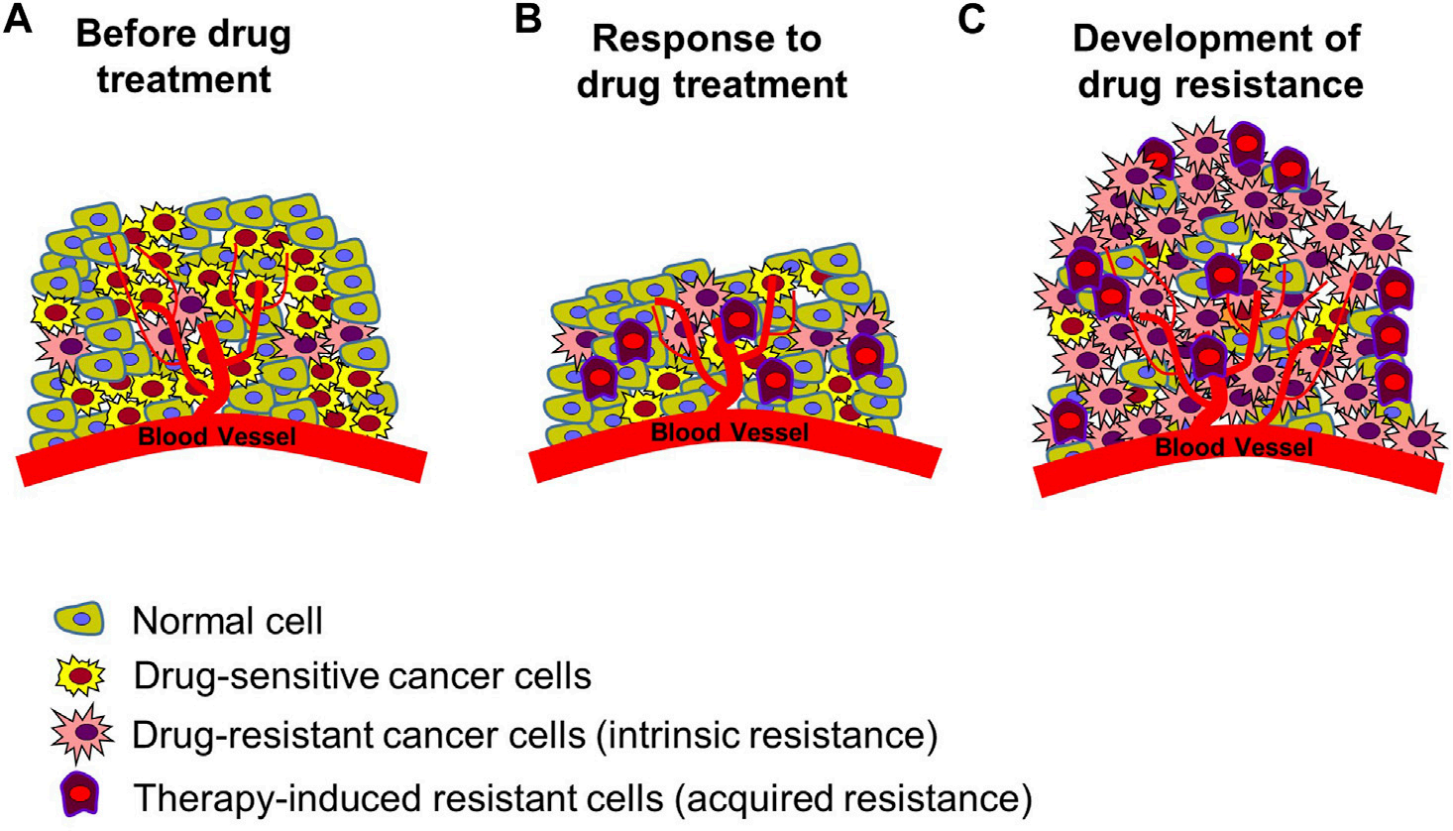
Development of resistance in tumors. **(A)** Tumor status prior to chemotherapy. There are normal cells, drug-sensitive cancer cells, and cancer cells with intrinsic resistance to many forms of treatment. **(B)** Induced cancer cell death following anticancer drug treatment. Drug-sensitive cancer cells die, existing resistant cells survive, and new therapy-induced resistant cancer cells are created. Reduction in size of cancerous tissue. **(C)** Enhanced anticancer drug resistance status. Increased proliferation of multiple types of resistant cells, leading to re-induced growth of cancerous tissue. Figure reproduced from Ref (Gottesman, 2002; Gottesman et al., 2002; Housman et al., 2014; Robey et al., 2018; Wang et al., 2019; Shi et al., 2022).

In the treatment of cancer, anticancer drug resistance refers to the development of chemotherapeutic agent resistance in cancer cells, which indicates that the cancer cells have not died despite receiving blood levels of the drugs that can kill drug sensitive cancer cells (Housman et al., 2014; Wang et al., 2019) Drug resistance can be categorized into two main types: intrinsic resistance, in which cancer cells are naturally resistant to a drug and do not respond to the drug, and acquired resistance, in which a drug treatment is initially effective in treating cancer but is continued in such a way that it results in numerous molecular alterations or induces the abnormal activity of other signaling systems, resulting in the drug no longer being effective (Figure 2) (Robey et al., 2018; Wang et al., 2019; Shi et al., 2022). Drug resistance arises from a range of molecular and cellular processes that are still largely unknown, including genetic mutations, epigenetic modifications, and cellular changes (Housman et al., 2014; Robey et al., 2018; Shi et al., 2022). Mechanisms of drug resistance identified till date include increased drug excretion due to overexpression of ATP binding cassette transporters (resulting in increased drug efflux and decreased drug accumulation) (Gottesman et al., 2002; Vadlapatla et al., 2013; Wu et al., 2014; Alfarouk et al., 2015; Robey et al., 2018; Shi et al., 2022), alterations of drug targets (e.g., target protein mutation, activation of alternative pathways to bypass the drug effect) (Bell et al., 2005; Ma et al., 2011; Wang et al., 2012; Tang et al., 2013; Housman et al., 2014; Wang et al., 2019), metabolic inactivation of drugs (Cumming et al., 2001; Townsend and Tew, 2003; Sampath et al., 2006; Zahreddin and Borden, 2013; Housman et al., 2014), epigenetics (epigenetic modifications can alter the expression of drug resistance-related genes) (Wu et al., 2014; Ohata et al., 2017), activation of DNA damage repair (reducing the anticancer drug effect that kills cancer cells by inducing DNA damage) (Esteller, 2000; Olaussen et al., 2006; Curtin, 2012), regulation of apoptotic pathways blocking cell apoptosis (increasing cancer cell survival by blocking programmed cell death induced by drugs) (Frew et al., 2008; Sasaki et al., 2010; Soria et al., 2010), epithelial-mesenchymal transition, tumor microenvironment (changes in the tumor environment can increase cancer cell protection by immune system from anticancer drug attack and reduce drug uptake through hypoxia condition) (Carraway and Sweeney, 2006; Lenisak et al., 2009; Singh and Settleman, 2010; Wendt et al., 2010), and cancer cell heterogeneity (Campbell et al., 2010; Navin et al., 2010; Parkin et al., 2013).

Despite significant research efforts, achieving desirable chemotherapeutic effects in the treatment of advanced cancer with single-drug therapy alone is considered nearly impossible due to the molecular complexity of cancer and its resistance to anticancer drugs. As a result, multi-targeted approaches have been adopted recently to enhance the effectiveness of chemotherapy and avoid drug resistance (Falzone et al., 2018; Delou et al., 2019). However, this is not a fundamental solution for overcoming anticancer drug resistance. Currently, drug therapy for cancer treatment primarily involves chemotherapy, and targeted drug therapy is under development. However, targeted therapy alone has not been able to overcome cancer drug resistance. Additionally, while immunotherapy, which involves directly engaging the patient’s immune system, has been attempted recently, it still faces limitations in inducing resistance and does not directly overcome cancer drug resistance (Said and Ibrahim, 2023). Therefore, for effective cancer treatment, overcoming drug resistance is an obstacle that requires a dramatic shift in our approach. Finding innovative strategies to tackle drug resistance is essential to improving the outcomes of cancer therapy.

## 5 Possible role of primary cilia in anticancer drug resistance

**FIGURE 3:**
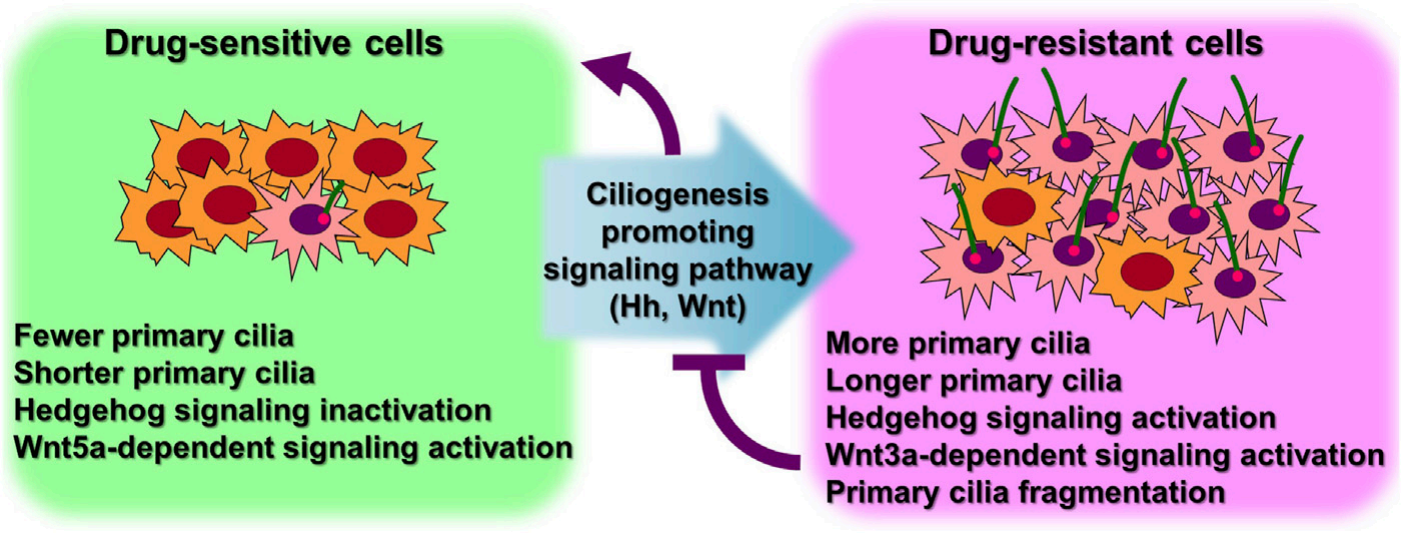
More ciliated status leads to anticancer drug resistance. Excessive primary ciliogenesis due to overactivation of ciliogenesis pathways leads to the acquisition of drug resistance in cancer cells. Both intrinsic and acquired resistance increase the length of primary cilia and the number of cells with primary cilia. Specific inhibition of primary ciliogenesis pathways overcomes drug resistance. Figure reproduced from Ref. (Jenks et al., 2018; Kyun et al., 2020; Kim et al., 2022).

Numerous cancer cell types have been shown to lack primary ciliogenesis (Eguether and Hahne, 2018; Higgins et al., 2019). Additionally, the primary cilia that grew when these cancer cells were exposed to anticancer drugs to create resistance were discovered to be very lengthy (Figure 3) (Table 1) (Jenks et al., 2018; Kyun et al., 2020; Kim et al., 2022). In cancer cells that were resistant to the anticancer drug kinase inhibitors, more cells generated primary cilia, and these cells’ primary cilia were longer than those of cancer cells that were not drug-resistant, according to a recent study. Tanos and associates (Jenks et al., 2018) looked at the relationship between primary cilia and drug resistance in HCC4006 lung adenocarcinoma cells that were resistant to the family of kinase inhibitors, erlotinib. Drug-sensitive HCC4006 lung adenocarcinoma cells do not form primary cilia under the 48-h serum starvation conditions required to induce primary cilia formation, but erlotinib-resistant HCC4006 cells were found to form primary cilia successfully when primary cilia formation was induced by 48 h serum starvation. They also found that, when compared to control NCI-H2228 cells, NVP-TAE684 (a small molecule ALK inhibitor)-resistant NCI-H2228 lung adenocarcinoma cells had more primary ciliated cells and longer primary cilia. When compared to control A204 cells, rhabdoid tumor A204 cells that developed dasatinib (a tyrosine kinase inhibitor) resistance had longer primary cilia and more ciliary tip fragmentation. These findings allowed them to predict how primary cilia induce drug resistance. They transfected A204 cells with Kif7 siRNA to promote elongated primary cilia generation via Kif7 knock-down to verify this theory, and they discovered that datatinib-induced cell death was reduced in these cells. Collectively, these findings imply that the development of drug resistance requires an increase in primary cilia length. Tanos and others (Jenks et al., 2018) then investigated the effects of drug resistance on primary ciliary inducible cell signaling. In dasatinib-resistant A204 cells compared to control A204 cells, SHH or SMO agonist (SAG) stimulation was able to induce enhanced GLI1 and PTCH1 mRNA expression, which are Hh target genes. They also discovered a comparable rise in the activity of Hh signaling in H2228 cells and HCC4006 cells resistant to the ALK inhibitor NVP-TAE684. They tested whether cancer cell survival was impacted by primary ciliogenesis suppression by knocking down either the IFT-B particle IFT88 or the centriole distal appendage protein SCLT1. The findings showed that erlotinib treatment of ErloR-HCC4006 cells sharply decreased cell viability through the prevention of primary ciliogenesis in ErloR-HCC4006 cells by reduction of IFT88 or SCLT1. This suggests that by inhibiting primary ciliogenesis, erotinib-resistant HCC4006 cells were likely converted to erlotinib-sensitive HCC4006 cells. Dasatinib-resistant A204 cells (DasR-A204) and NCI-H2228 NVP-TAE684-resistant cells had comparable outcomes. Together, Tanos and her colleagues demonstrate that primary cilia are essential for the development of drug resistance and that they can serve as a target for combating it. Later research has strengthened the link between primary cilia and drug resistance.

**TABLE 1:** Primary cilia and drug resistance in cancer cell lines.

Cancer cell lines	Drug resistance	Primary cilia length	Cells with primary cilia	References
A549 (Lung carcinoma)	Doxorubicin	increase	increase	Kim et al. (2022)
	Paclitaxel	increase	increase	Kim et al. (2022)
	Dasatinib	increase	-	Kim et al. (2022)
	Trametinib	increase	increase	Jenks et al. (2018)
	Cisplatin	increase	-	Jenks et al. (2018)
	Carboplatin	-	-	Jenks et al. (2018)
	Vinflunine	increase	increase	Jenks et al. (2018)
H2228 (Lung adenocarcinoma)	NVP-TAE	increase	increase	Jenks et al. (2018)
HCC4006 (Lung adenocarcinoma)	Erlotinib	increase	increase	Jenks et al. (2018)
PC-9 (Lung adenocarcinoma)	Afatinib	-	-	Jenks et al. (2018)
	Erlotinib	-	-	Jenks et al. (2018)
H23 (Lung adenocarcinoma)	Trametinib	increase	increase	Jenks et al. (2018)
H1792 (Lung adenocarcinoma)	Trametinib	-	increase	Jenks et al. (2018)
A204 (Muscle rhabdomyosarcoma)	Dasatinib	increase	-	Jenks et al. (2018)
PANC-1 (Pancreatic ductal adenocarcinoma)	Cisplatin	N.D.	increase	Chao et al. (2022)
MCF-7/ADR (Multidrug-resistant breast tumor cell line)	Doxorubicin (Adriamycin)	increase	increase	Kyun et al. (2020)

N.D., not determined.

Lee and his colleagues (Kyun et al., 2020) identified a pathway through which Wnt3a, one of the canonical Wnt ligands, causes primary ciliogenesis. They discovered that Wnt3a stimulation increased the number of cells generating primary cilia, and Wnt3a stimulation enhanced primary cilia length and thickness. When Wnt3a was stimulated, CK1δ was activated, which phosphorylated β-catenin S47. It was later discovered that β-catenin p-S47 is located in the mother centriole’s subdistal appendage and that it causes the centriolar satellites PCM1, AZI1, and CEP290 to accumulate centrosomally. Primary cilia were induced to grow as a result of this chain of events. They tested the MCF-7/ADR cells, breast cancer-derived adriamycin (doxorubicin)-resistant cells, using this pathway established in hTERT-RPE cells, and observed that it was highly active in MCF-7/ADR cells (Kyun et al., 2020). In comparison to control MCF-7 parental cells, Wnt3a stimulation of MCF-7/ADR cells increased primary cilia generation and length by an average of 7 and 3 folds, respectively. Compared to the roughly 2-fold increase in primary ciliated cells and primary ciliary length shown in hTERT-RPE cells without drug resistance, this rise is much larger. Additionally, drug-resistant MCF-7/ADR cells showed a 7-fold increase in centrosomal accumulation of centriolar satellites in response to Wnt3a stimulation compared to controls, whereas only a 2-fold increase was seen in hTERT-RPE cells. These findings suggest that drug-resistant cells are more likely to have active primary ciliogenesis through the Wnt3a stimulation mechanism than normal cells. Another study confirmed that the induction of anticancer drug resistance promotes primary ciliogenesis in anticancer drug-resistant A549 cells by treating the lung cancer cell line with doxorubicin, dasatinib, and paclitaxel to induce resistance (Kim et al., 2022). The extent of primary cilia formation was then assessed. Primary ciliated cells and primary ciliary length were both enhanced in the doxorubicin- and paclitaxel-resistant A549 cells (A549/Dox and A549/Pac, respectively) in comparison to parental A549 cells. Cells that were resistant to dasatinib showed somewhat varied outcomes. The number of primary ciliated cells in dasatinib-resistant A549 cells (A549/Das) was not substantially different from parental A549 cells, but they had increased primary ciliary length and fragmentation. These findings demonstrate a robust relationship between primary ciliogenesis and drug resistance, and the potential of primary cilia as a target for overcoming drug resistance in cancer therapy.

## 6 Perspectives

**FIGURE 4:**
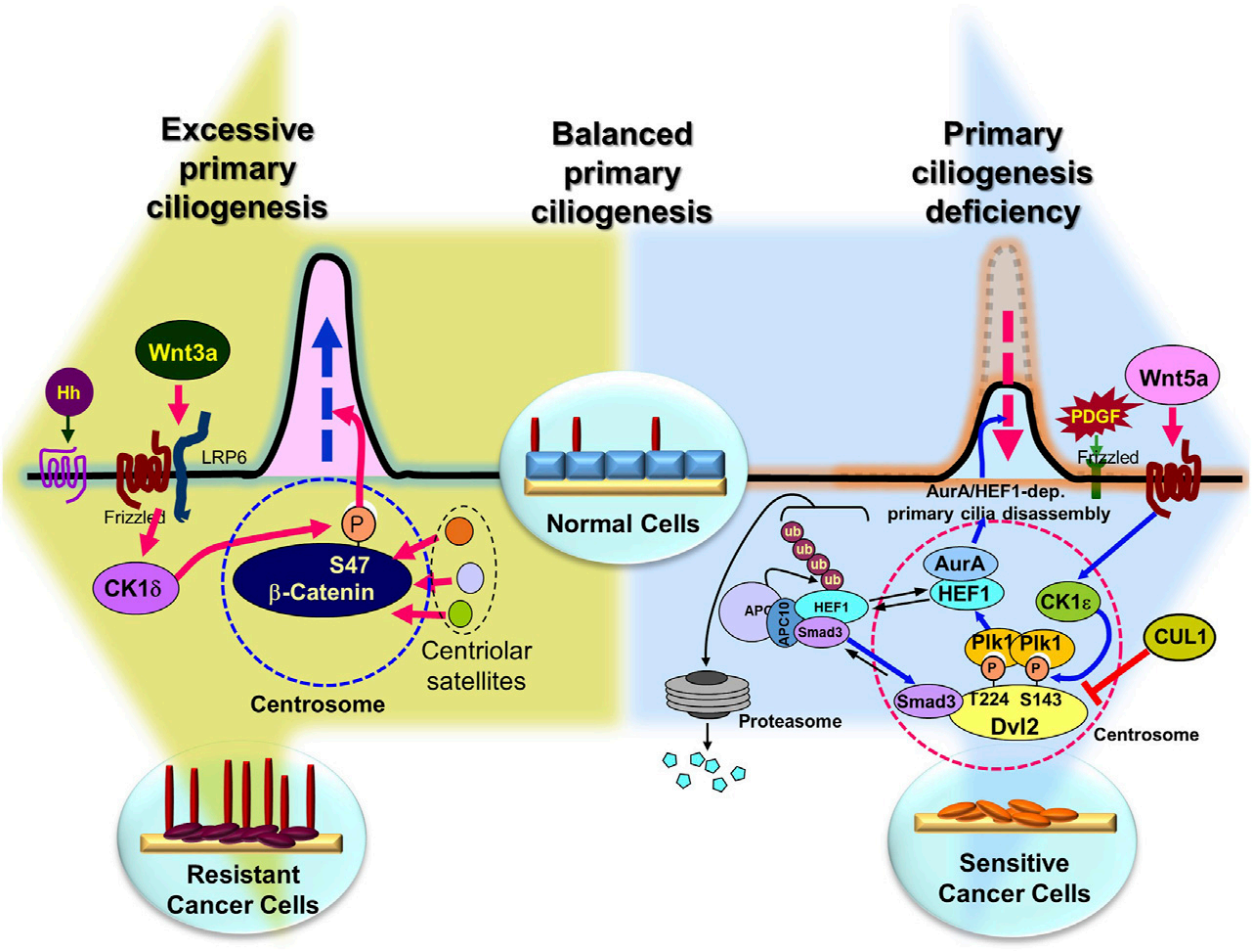
Overcoming anticancer drug resistance by regulating primary ciliogenesis in cancer cells. Presumably, in normal cells, the right amount/length of primary cilia are formed at the right time, but overactivation of the primary cilia disassembly pathway induces extreme primary cilia loss (large blue background arrow), leading to cancer (although there are exceptions), and in cancer cells, overactivation of the primary cilia assembly pathway leads to the development of drug resistance (large pea-green background arrow). Therefore, it is possible to inhibit tumorigenesis by inhibition of excessive primary cilia disassembly pathway or induction of assembly pathway, and to overcome drug resistance by inhibition of primary cilia assembly pathway or activation of disassembly pathway in anticancer drug-resistant cells. Figure reproduced from Ref (Pugacheva et al., 2007; Lee et al., 2012; Jenks et al., 2018; Kyun et al., 2020; Lee, 2020; Kim et al., 2021; Kim et al., 2022).

Despite decades of research, cancer drug resistance has not been overcome. Recently, targeted and immunotherapeutic drugs have been used to combat the condition, although drug resistance has also been discovered (Said and Ibrahim, 2023). Therefore, overcoming cancer drug resistance requires a breakthrough approach, not an iteration of current treatments. This review suggests that primary cilia are the cellular organelles that underlie drug resistance and are potential targets for overcoming anticancer drug resistance (Figure 4). The role of primary cilia has been overlooked in favor of the function of motile cilia (Davenport and Yoder, 2005). However, its importance has recently been acknowledged because of several reports of linkages to human illness. Its position as a center of cellular signaling that maintains cellular homeostasis has made it a key organelle in associated illnesses (Goetz and Anderson, 2010; Seeley and Nachury, 2010). Recent evidence shows that primary cilia manipulation may potentially be used to overcome anticancer drug resistance (Figure 4) (Table 1) (Jenks et al., 2018; Kyun et al., 2020; Kim et al., 2022). However, the importance of primary cilia in drug resistance is not entirely known, and research on the role of primary cilia in drug resistance continues. The general application of primary ciliary-related drug resistance mechanisms to each cancer type needs further investigation, as does the varied primary ciliary effects in different cancer types. The current results are sufficient to shift the focus from the limitations of traditional drug resistance research to overcome resistance through primary cilia research. Therefore, it is crucial to identify key factors that are specifically involved in the primary cilia production regulation, degradation, and function to develop targets to overcome anticancer drug resistance with minimal side effects and toxicity. Demonstrating that the identified primary cilia-specific factors are connected to anticancer drug resistance may provide a breakthrough and a new tool to overcome anticancer drug resistance using primary cilia. Furthermore, since primary cilia are deeply involved in the cancer microenvironment (Bailey et al., 2008; Shaw et al., 2009; O’Toole et al., 2011; Liu et al., 2018; Wang et al., 2021) primary cilia research targets may provide a strategy for overcoming cancer drug resistance while also modulating the cancer microenvironment.
